# Advances in Imaging-Based Fracture Risk Assessment for Unlocking Latent Skeletal Fragility

**DOI:** 10.1007/s11914-026-00961-6

**Published:** 2026-03-21

**Authors:** Yisak Kim, Sung Hye Kong

**Affiliations:** 1 Interdisciplinary Program in Bioengineering, Graduate School, Seoul National University, Seoul, South Korea; 2 Department of Radiology, Seoul National University Hospital, Seoul, South Korea; 3 Division of Endocrinology and Metabolism, Department of Internal Medicine, Seoul National University Bundang Hospital, Seongnam, South Korea; 4 Department of Internal Medicine, Seoul National University Bundang Hospital, Seoul National University College of Medicine, 82, Gumi-Ro 173 Beon-Gil, Bundang-Gu, Seongnam, Gyeonggi-Do 13620 Republic of Korea

**Keywords:** Osteoporosis, Fracture Risk Assessment, Opportunistic Screening, Deep Learning

## Abstract

**Purpose of Review:**

This review summarizes recent advancements in imaging-based fracture risk assessment utilizing routinely acquired clinical images. We explore how imaging-derived methodologies and deep learning techniques can enhance conventional tools, such as dual energy X-ray absorptiometry (DXA)-derived bone mineral density and FRAX®, by capturing additional factors influencing skeletal fragility.

**Recent Findings:**

Recent studies indicate that opportunistic analyses of computed tomography, radiographs, DXA, and magnetic resonance imaging facilitate the estimation of bone density, the detection of previously unrecognized vertebral fractures, and the extraction of biomarkers associated with bone quality, muscle composition, and skeletal geometry. Additionally, recent research demonstrates that end-to-end deep learning models can directly predict future fracture risk from raw images across various imaging modalities.

**Summary:**

Imaging-based approaches reveal that clinically relevant fracture risk information is embedded within routine clinical images beyond traditional measurements. These methods have the potential to mitigate gaps in fracture risk assessment and support scalable prevention strategies. Further research is necessary to enhance robustness and facilitate clinical integration.

## Introduction

Fragility fractures represent a significant source of morbidity and mortality among older adults [1–3]. Dual energy X-ray absorptiometry (DXA)-derived bone mineral density (BMD) and fracture risk assessment tools, such as FRAX®, are critical for evaluating fracture risk, with substantial evidence affirming their clinical relevance [4–6]. Nonetheless, numerous prospective cohort studies consistently reveal that a significant proportion of fractures occur in individuals who do not exhibit densitometric osteoporosis [7, 8]. This discrepancy underscores the inherently multifactorial nature of skeletal fragility, which is influenced by structural and biomechanical factors that extend beyond the scope of these traditional measures [9, 10]. DXA-derived BMD offers a low-dimensional representation of skeletal integrity, invariably compressing complex spatial, geometric, and textural information into a singular scalar value [11]. As a result, key factors contributing to fracture susceptibility—such as undetected vertebral fractures [12], deterioration of trabecular and cortical architecture [13], altered geometry [14], and age-related muscle decline [15]—are often overlooked in routine evaluations.

Simultaneously, vast quantities of computed tomography (CT), X-ray, and magnetic resonance imaging (MRI) data are routinely generated in clinical practice [16]. These imaging modalities serve as high-dimensional sensors, capturing latent structural and functional information about bone, muscle, and surrounding tissues that is frequently disregarded when imaging is interpreted solely for its primary diagnostic intent [17]. Recent advancements in imaging artificial intelligence (AI) and machine learning enable the quantification of these subtle cues opportunistically, without the need for additional scans or costs, thereby complementing traditional assessment tools [18]. Imaging-derived biomarkers can unveil dimensions of bone quality, muscle status, and structural vulnerability that may enhance existing risk stratification frameworks [19–21].

To organize this rapidly evolving research landscape, this review synthesizes imaging-based fracture risk assessment approaches into four complementary domains: 1) imaging-derived BMD or osteoporosis detection; 2) automated identification of prevalent vertebral fractures for patient risk stratification; 3) extraction of structural and functional imaging biomarkers beyond fractures, including measures of muscle quality, bone microarchitecture, and geometry; and 4) end-to-end deep learning models that infer future fracture risk assessments directly from raw images.

**Fig. 1:**
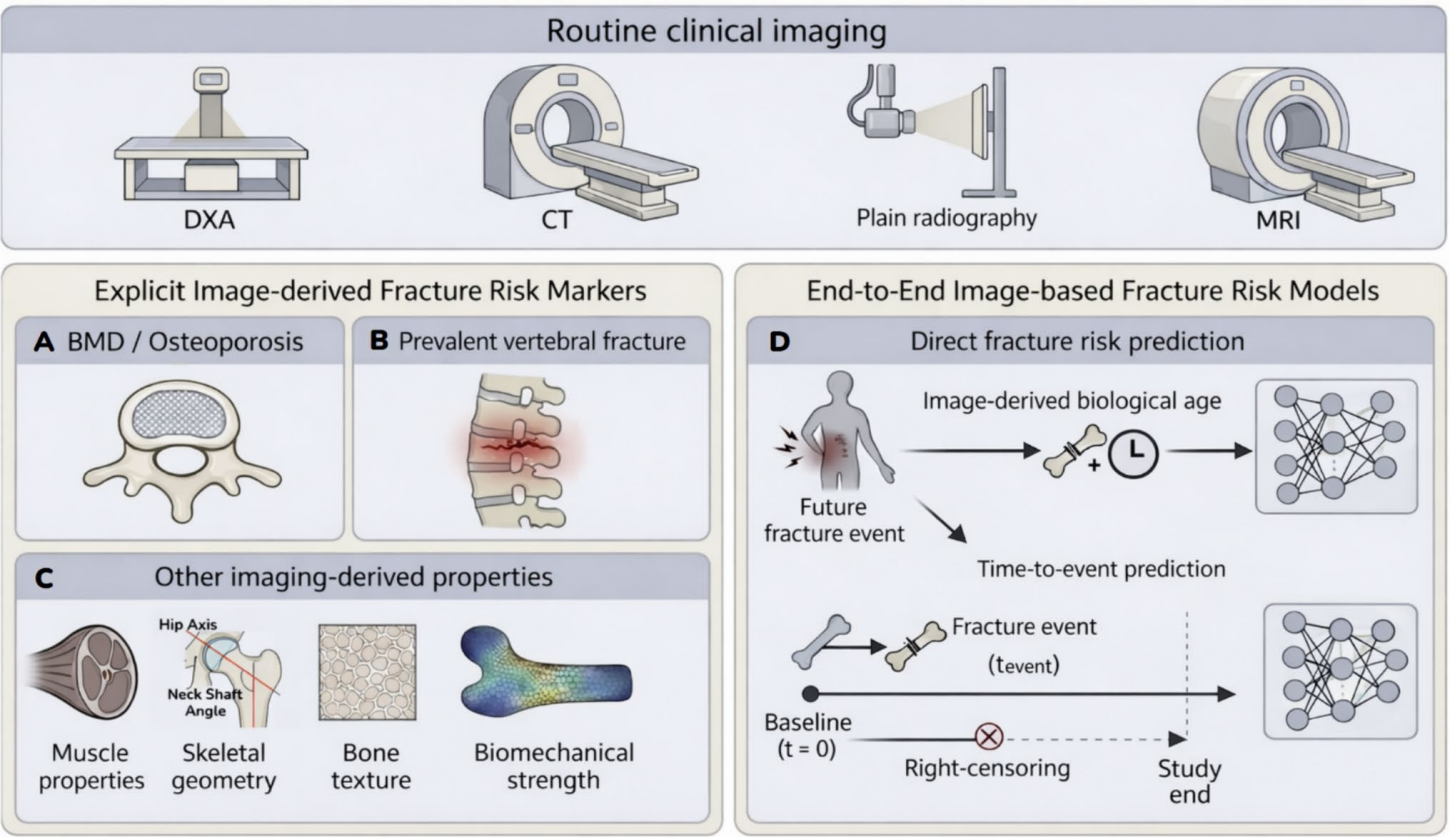
Overview of imaging-based fracture risk assessment approaches using routine clinical images. (A–C) Explicit image-derived fracture risk markers: Deep learning models extract specific biological features such as (**A**) BMD or osteoporosis status, (**B**) prevalent vertebral fractures, and (**C**) other properties including muscle composition, skeletal geometry, bone texture, and biomechanical strength. (**D**) End-to-end image-based fracture risk models: Deep learning algorithms directly infer future fracture risk from raw images using representation learning or time-to-event survival frameworks, without relying on predefined measurements

From a methodological standpoint, these domains can also be characterized by their underlying deep learning task formulations and learning objectives. The majority of existing studies can be categorized into several broad areas: image-based regression or classification models that approximate established clinical metrics such as BMD or osteoporosis status; detection or classification models aimed at identifying prevalent fractures or structural abnormalities; feature-extraction approaches designed to quantify specific imaging biomarkers related to bone quality, muscle composition, or skeletal geometry; and end-to-end prognostic models that predict future fracture events, often employing time-to-event or survival learning frameworks (Fig. 1).

These methodological categories vary not only in network architecture and supervision strategies but also in their intended clinical applications—ranging from opportunistic screening and risk enhancement to automated longitudinal risk stratification (Table 1). Explicitly distinguishing these approaches facilitates clearer interpretation of reported performance and aids in identifying the clinical contexts in which each method may be most effectively applied. Collectively, these methodologies illustrate how routinely acquired clinical images, when combined with modern analytical techniques, can help bridge persistent gaps in fracture risk assessment and support more comprehensive prevention strategies [22].

**Table 1:** Deep learning methodologies and clinical tasks in imaging-based fracture risk assessment

Clinical Task	DL Methodology	Learning Objective	Representative Clinical Use
Opportunistic BMD estimation/osteoporosis detection	Regression/Classification	Predict BMD surrogate or osteoporosis status	Risk enrichment in patients without DXA
Vertebral fracture detection	Detection/Classification	Identify prevalent fractures	Identify high-risk individuals for secondary prevention
Extraction of structural or compositional biomarkers	Radiomics + ML/CNN feature extraction	Quantify muscle, fat, texture, geometry	Capture non-BMD contributors to fragility
Direct prediction of future fracture risk (fixed horizon)	Classification/regression	Predict incident fracture within a predefined period	Opportunistic fracture screening
Time-to-event fracture risk prediction	Deep survival models (e.g., hazard-based, time-to-event networks)	Model fracture risk over time with censoring	Personalized longitudinal risk stratification

BMD, bone mineral density; DXA, dual-energy x-ray absorptiometry; ML, machine learning; CNN, convolutional neural network

### Imaging-based BMD Analysis or Osteoporosis Detection for Fracture Prediction

An initial focus in imaging-based fracture prediction has been the utilization of routinely acquired clinical images to derive quantitative surrogates of BMD or to opportunistically identify osteoporosis. This approach arose from the recognition that CT, DXA, and radiographic images inherently contain information about bone mineralization that is often overlooked in routine clinical practice [23]. Consequently, researchers began to evaluate whether attenuation-based measures or density surrogates extracted from standard clinical imaging could replicate—or enhance—the prognostic value of DXA-derived BMD.

Early opportunistic CT studies established a foundation by demonstrating that vertebral trabecular attenuation on abdominal or thoracic CT scans is strongly associated with future fractures. Pickhardt et al. illustrated that CT-derived attenuation could predict long-term major osteoporotic fractures with a performance superior to that of the FRAX® tool. Based on their foundational work, specific L1 attenuation thresholds were established for opportunistic screening: a threshold of 160 HU or less demonstrated 90% sensitivity for distinguishing osteoporosis, while a threshold of 110 HU provided more than 90% specificity. Furthermore, this approach proved highly valuable in identifying DXA false-negative results; an attenuation threshold of 145 HU or less successfully captured 97% of patients who had moderate-to-severe vertebral fractures despite having nonosteoporotic DXA T-scores [24, 25]. Extending this to longitudinal risk prediction, Lee et al. showed in real-world practice that a simple measurement of HU at the L1 vertebra could effectively stratify incident fracture risk. Rather than relying strictly on diagnostic sensitivity and specificity, they proposed an intuitive clinical threshold of approximately 110 HU, below which the hazard ratio (HR) for future osteoporotic fractures increases significantly, thereby facilitating broader clinical adoption [26]. The appeal of such methodologies lies in their simplicity, interpretability, and direct connection to well-established physical principles, which likely contributed to their early translational success [27].

Building on these findings, subsequent research expanded from single-level attenuation metrics to more comprehensive population-level analyses. Dagan et al. incorporated features from chest and abdominal CT into a machine-learning framework, achieving system-wide fracture prediction that matched or surpassed traditional tools [21]. This evolution, from basic HU thresholds to multifactorial models, reflects a growing interest in leveraging the full informational richness of CT imaging. Parallel studies extended opportunistic BMD estimation to modalities such as cardiac CT, demonstrating that even non-skeletal imaging can yield fracture-relevant density metrics [28]. Further enhancement was achieved through biomechanical approaches. Large-scale longitudinal studies, such as Li et al.’s involving a multiethnic cohort, confirmed the long-term prognostic significance of thoracic trabecular BMD for hip and vertebral fractures, even when accounting for baseline vertebral fractures [29].

Collectively, across various methodologies, imaging-derived BMD metrics consistently provide added value when combined with clinical predictors such as FRAX®, reinforcing their role as complementary rather than substitutive tools. Simultaneously, these approaches highlight important technical limitations, including sensitivity to scanner calibration, acquisition parameters, and reconstruction kernels, which necessitate ongoing development of harmonization strategies and more flexible modeling frameworks [30]. Recent advancements in deep learning further this trajectory by capturing complex spatial patterns related to bone quality and microarchitecture that are not represented by conventional measurements.

### Imaging-based Fracture Detection for Future Fracture Prediction

A second focus in imaging-based fracture prediction involves identifying previously unrecognized vertebral fractures (VFs) and utilizing them as significant prognostic indicators for future fractures. Prevalent VFs often remain undetected in routine clinical workflows, despite their status as some of the strongest predictors of subsequent fracture events. Rather than being viewed as isolated diagnostic targets, prevalent VFs can be conceptualized as observable manifestations of an underlying skeletal failure state that accumulates over time [31]. With the growing availability of CT, spine radiographs, and DXA-based vertebral fracture assessment (VFA), automated detection methods have been developed to systematically reveal these hidden fractures and integrate them into risk prediction models.

Early studies have established that opportunistically detected vertebral deformities in non-skeletal imaging possess significant prognostic implications. A pivotal investigation by Buckens et al. demonstrated that VFs incidentally identified on chest CT scans were associated with a markedly increased risk of subsequent hip fractures (HR 3.1). This finding underscores that unreported fractures captured during routine imaging can effectively stratify fracture risk [32]. In a similar vein, Skjødt et al. analyzed routine CT scans within a real-world cohort and observed that untreated individuals with CT-identified VFs experienced significantly higher subsequent fracture rates over a seven-year period [33], thereby reinforcing the clinical consequences of overlooked VFs in everyday practice.

Recent advancements in deep learning have further broadened the scope and scalability of VF detection. Hong et al. developed a multitask model capable of identifying vertebral fractures from spine X-ray and DXA-VFA images, demonstrating strong prognostic value across two large cohorts: HR 3.23 in the development cohort and HR 2.11 in the validation cohort [19]. These results indicate that automated detection serves not only as a diagnostic tool but also as a prognostic instrument, effectively identifying individuals at significantly elevated long-term risk. Likewise, Kong et al. utilized opportunistic CT-derived features to predict VFs over a five-year horizon, with both internal and external validation confirming generalizability across various imaging environments [20].

Overall, these investigations illustrate a coherent trajectory in which the automated identification of VFs has evolved from incidental findings on chest CT to robust, AI-driven detection systems suitable for large-scale deployment. Importantly, this domain emphasizes the conceptual distinction between detection accuracy and prognostic relevance; even imperfect detection models can provide substantial clinical utility if they consistently identify individuals at heightened risk of future fractures.

### Imaging-based Detection of Additional Factors Related to Fracture Risk

Beyond bone density and explicit fracture detection, a significant area of advancement in imaging-based fracture prediction is the quantification of additional structural and functional determinants of skeletal fragility using routine imaging techniques. Given that fracture risk results from the interplay among bone quality, muscle performance, fat infiltration, and mechanical loading, these studies utilize CT, DXA, X-ray, and MRI to extract imaging biomarkers that reflect these broader contributors to bone strength [18, 34, 35]. Collectively, this body of work represents a hypothesis-driven, feature engineering paradigm in which specific imaging-derived measurements are designed to capture the presumed mechanisms underlying fracture susceptibility. This approach acknowledges that incident fractures frequently occur in individuals without densitometric osteoporosis and seeks to elucidate physiologic vulnerabilities that are not represented by BMD alone.

One primary focus of this research has been the assessment of muscle and adipose compartments. In early prospective studies, Sheu et al. demonstrated that reduced psoas muscle volume and increased intramuscular adipose tissue observed on abdominal quantitative CT were independently associated with incident non-spine fractures, even after adjusting for BMD [36]. Harvey et al. later confirmed that lower muscle density, as assessed by peripheral quantitative CT, is a significant predictor of major osteoporotic fractures across international datasets [37]. These findings highlight the importance of sarcopenic and lipotoxic changes in fracture susceptibility, supporting a triadic perspective of bone, muscle, and fat interplay.

Other studies have evaluated image-derived indicators of bone quality and texture. Utilizing DXA hip images, Hong et al. derived radiomic signatures that independently predicted incident hip fractures, surpassing the predictive capabilities of FRAX® and BMD [38]. Similarly, Thevenot et al. demonstrated that pelvic X-ray texture features could effectively classify hip fracture risk, suggesting that subtle architectural variations visible on low-dose projection images reflect clinically meaningful skeletal fragility [39]. These radiomic approaches demonstrate that conventional imaging contains latent microarchitectural patterns that contribute to the risk of skeletal failure.

Assessing microarchitectural deterioration is another critical feature for understanding skeletal fragility. Trabecular Bone Score (TBS) represents a major, widely adopted advancement in this domain. As a gray-level textural metric derived directly from routine lumbar spine DXA images, TBS provides a surrogate measure of bone microarchitecture that is not captured by standard areal BMD [40, 41]. Extensive clinical validation has demonstrated that a degraded TBS is an independent predictor of major osteoporotic and vertebral fractures, effectively identifying at-risk individuals who might not meet the densitometric criteria for osteoporosis [42]. Consequently, TBS has been successfully integrated into clinical practice as an adjustment factor for the FRAX® algorithm, thereby enhancing the precision of fracture risk stratification without requiring additional radiation exposure or specialized imaging [43].

While TBS evaluates internal trabecular texture, the overall mechanical competence and skeletal geometry have also been explored through hip structural analysis and finite element modeling. A study by Kaptoge et al. revealed that geometric indices could predict long-term hip fractures independently of BMD [44]. Subsequent studies utilizing DXA-derived finite element (FE) strength, including those by Yang et al., demonstrated superior or complementary predictive performance relative to FRAX® and BMD [45]. This trajectory continued with CT-based biomechanical assessments: Adams et al. showed enhanced sensitivity for hip fracture prediction through FE strength derived from routine body CT [46], while multiethnic longitudinal studies by Fleps [47] and Praveen et al. [48] confirmed that FE-based measures maintain predictive superiority for up to 16 years of follow-up. More recently, Grassi et al. reconstructed three-dimensional FE models from standard two-dimensional DXA images, achieving robust and independent predictions of 10-year hip fractures, thus suggesting broader feasibility in clinical practice without the resource burden associated with CT [49].

Collectively, the integration of microarchitectural indices like TBS with macroscopic biomechanical strength estimations from FE provides a comprehensive, multidimensional evaluation of skeletal fragility. These routine software-driven advancements demonstrate that both conventional DXA and CT scans contain critical structural information that extends far beyond simple densitometry, enabling a much more robust assessment of fracture risk.

MRI has further broadened the spectrum of measurable bone quality. Vertebral bone quality (VBQ) scores, assessed via non-contrast T1-weighted MRI, have been independently associated with fragility fractures in observational cohorts. Ehresman et al. reported that an elevated VBQ score is a significant independent predictor of fractures [50]. Rather than establishing a single diagnostic cutoff, they demonstrated that VBQ scores evaluated as a continuous variable significantly increased the odds of incident fracture, yielding an odds ratio of 2.40 for each one-point increase in the score. Furthermore, they observed significantly higher mean VBQ scores in patients who sustained fragility fractures compared to those who did not (3.50 vs. 3.01). Additionally, these MRI-based metrics have demonstrated temporal prognostic value, showing potential for the prediction of imminent new vertebral fractures within a two-year timeframe [51]. These MRI-based approaches are particularly valuable because they capture early changes in marrow adiposity and tissue composition that often precede measurable declines in BMD.

This expanding body of evidence illustrates that imaging can quantify a diverse array of skeletal and extraskeletal risk factors that reinforce and refine traditional assessments. Across various modalities and methodologies, these biomarkers consistently provide independent and additive value beyond BMD and FRAX®. As these tools continue to develop, the integration of multi-domain signals may facilitate a more comprehensive characterization of fracture susceptibility that more accurately reflects the biological complexity of skeletal aging.

### Imaging-based Direct Fracture Prediction (End-to-End Models)

A growing body of research has transitioned from using predefined density metrics and explicit fracture detection methods to directly predicting future fracture events from raw imaging data. This end-to-end paradigm allows deep learning models to infer fracture risk by utilizing the entire visual context of the image, thereby removing the need to rely on manually defined regions of interest or handcrafted features. Conceptually, this approach redefines fracture prediction as a representation learning problem, where fracture risk is encoded as a latent property of the image rather than derived from explicitly specified measurements. It capitalizes on the understanding that skeletal fragility is not solely represented by specific landmarks but also manifests in subtle, globally distributed patterns that may be imperceptible to the human eye.

Several studies have underscored the potential of this strategy across various modalities and clinical settings. For instance, using longitudinal total-body DXA imaging, deep learning models have successfully predicted fixed-horizon outcomes, including 10-year all-cause mortality [52] and hip fracture risk [53], demonstrating that serial imaging can facilitate long-term risk stratification without the need for explicit feature engineering. In patients with a history of hip fractures, Kim et al. utilized CT-derived digitally reconstructed radiographs to forecast subsequent fractures over a five-year period, achieving superior prognostic performance compared to traditional clinical models [54]. Kong et al. employed a time-to-event neural network on routine lateral lumbar radiographs, revealing that image-derived risk signals could predict incident VFs without prior fracture labels [55]. Chen et al. further highlighted the feasibility of broader deployment by developing a chest X-ray–based model that predicted five-year vertebral compression fractures with strong external validation, suggesting that commonly acquired thoracic imaging may facilitate opportunistic fracture screening [56]. End-to-end models have also been used to derive high-level biological signatures from images. Cho et al. introduced an image-based "Spine-Age" metric derived from lateral radiographs and VFA studies, which correlated with both fracture and mortality risk across multiple validation cohorts [57].

These studies illustrate that end-to-end image-based fracture modeling can be achieved through various deep learning formulations, each reflecting distinct engineering trade-offs in problem definition. Fixed-horizon prediction and risk regression approaches directly map imaging features to fracture outcomes within a predefined time window, offering simplicity and computational efficiency [52, 53]. In contrast, representation-focused models learn latent, image-derived embeddings that encapsulate cumulative skeletal vulnerability or biological aging, such as image-based age metrics, thereby allowing downstream associations with multiple clinical outcomes [57].

Time-to-event modeling frameworks further enhance these methods by explicitly incorporating follow-up duration and censoring into the learning objective, thereby preserving temporal information that is typically lost in fixed-time formulations [54–56]. Despite differences in output definition and optimization strategies, all these methods share a common engineering principle: they leverage raw imaging data to learn prognostically meaningful representations without reliance on predefined measurements, while differing in their treatment of temporal structure and their handling of uncertainty.

These investigations share a unifying goal: to extract the totality of latent information related to structural weakness, loading vulnerability, and systemic aging from routine imaging, without prespecifying which aspects of the image are most relevant. Advances in model interpretability, such as Grad-CAM and SHAP, have begun to reveal that neural networks often focus on trabecular-rich regions, cortical contours, and even extraskeletal tissues like paraspinal musculature, reinforcing the biological credibility of these approaches [54, 55]. Moreover, survival-based architectures that integrate longitudinal electronic health record (EHR) data are increasingly being adopted, aligning more closely with real-world clinical decision-making [58].

In summary, end-to-end imaging models represent a natural evolution in fracture risk assessment. By eliminating dependence on explicit measurements and leveraging comprehensive image information, these methods have the potential to enable truly opportunistic, scalable, and automated screening across diverse imaging environments. As integration with clinical workflows improves, this direction holds promise for bridging persistent diagnostic gaps and redefining how fracture risk is identified in routine care.

### Summary and Future Perspectives

Imaging-based fracture risk assessment has advanced significantly beyond the sole reliance on BMD, leading to a more nuanced characterization of skeletal fragility. By integrating markers of structural geometry, microarchitectural deterioration, biomechanical strength, and the broader bone–muscle–fat axis, recent studies reveal that clinically acquired imaging contains a wealth of underutilized information pertinent to fracture risk. This evolution signifies a shift from isolated, low-dimensional measurements toward integrative modeling of fracture vulnerability as a systemic phenomenon. Notably, end-to-end deep learning methodologies illustrate that susceptibility to fracture can be inferred directly from raw images, reconceptualizing fracture risk as a latent, image-encoded state rather than a value derived solely from predefined metrics. This suggests that fragility is interwoven throughout the visual landscape of skeletal and extraskeletal tissues, even when not captured by conventional measurements.

Despite these promising advancements, to realize the full potential of these evolving methodologies, future research must systematically resolve translational challenges across three main domains: technical barriers, data limitations, and clinical implementation. First, regarding technical barriers, a primary hurdle is multi-center applicability. Performing reliable fracture risk assessment and bone density prediction remains inherently difficult across different imaging modalities, manufacturers, and specific device types. Variations in hardware specifications, proprietary image reconstruction algorithms (e.g., kernels), and institutional acquisition protocols introduce significant 'domain shifts' into the imaging data. Consequently, AI models trained predominantly on data from a single center or a specific scanner type frequently experience reduced predictive performance when deployed in external validation cohorts. To ensure reproducibility and facilitate large-scale clinical integration, future research must prioritize the development of scanner-agnostic foundation models and the application of robust cross-institutional image harmonization strategies.

This challenge of hardware and protocol heterogeneity is particularly pronounced in projectional imaging modalities like routine chest radiographs. Variations in soft tissue thickness, composition, and lung aeration create substantial superimposition artifacts that can obscure true bone texture. Furthermore, unlike quantitative CT or DXA, routine chest X-rays lack standard calibration phantoms. Consequently, inconsistencies in imaging conditions—such as variations in tube voltage (kVp), exposure time (mAs), source-to-image distance, and patient positioning—can drastically alter the apparent radiopacity of bone, thereby confounding deep learning models. Addressing these modality-specific barriers necessitates the development of robust, physics-informed image harmonization techniques alongside a strong consensus on standardizing image quality across varying equipment manufacturers.

A critical clinical manifestation of these technical and modality-specific barriers is the current lack of consensus on universally applicable diagnostic cutoff values for imaging biomarkers. While specific risk-stratifying thresholds—such as 110 HU for CT—have demonstrated strong prognostic utility in isolated studies, these absolute values remain highly vulnerable to imaging conditions. Raw CT attenuation is profoundly influenced by variations in tube voltage (kVp), scanner calibration, and patient size, whereas MRI VBQ scores, despite internal referencing to cerebrospinal fluid, can still fluctuate depending on magnetic field strength (1.5 T vs. 3.0 T) and coil performance. Therefore, directly applying a rigid cutoff or reference range across different institutions without calibration is fundamentally flawed. To establish universally reliable metrics, future research must shift toward the development of asynchronous, phantom-less calibration methods (e.g., using patient-specific internal reference tissues like muscle or fat) and standardized, AI-driven signal normalization protocols that mathematically adjust for hardware-induced variations prior to risk calculation.

Second, beyond technical standardization, significant data and validation limitations remain. The long-term outcome data necessary for training and validating prognostic models are challenging to obtain, necessitating large-scale, well-curated longitudinal cohorts. Finally, true clinical implementation and workflow integration pose significant issues related to electronic health record (EHR) interoperability, regulatory approval, and clinical accountability. Furthermore, rigorous health-economic evaluations will be essential to justify the cost-effectiveness of these opportunistic assessments in routine practice. All of these multifaceted challenges must be resolved to realize scalable and precision-oriented fracture prevention.

Looking ahead, various developments are set to accelerate progress. The integration of imaging with clinical, biomechanical, and omics data may provide a more comprehensive representation of skeletal aging. Foundation models and self-supervised learning can leverage the extensive volumes of unlabeled clinical images to enhance model robustness and diminish reliance on outcome-rich datasets. Harmonization strategies can facilitate privacy-preserving collaboration across institutions.

Viewed together, these advancements suggest a future where fracture risk is derived less from manually engineered metrics and more from holistic, data-driven signatures learned directly from images routinely acquired in clinical practice. In this framework, fracture susceptibility is not inferred from a limited set of predefined measurements but from integrated image-level representations that inherently capture structural integrity, biomechanical competence, and extraskeletal factors contributing to failure risk. As methodologies continue to evolve and align more closely with longitudinal outcomes, end-to-end imaging analysis is poised to play a central role, bringing us closer to timely, scalable, and precision-oriented fracture prevention.

## Key References

- 19. Hong, N., et al., Deep learning-based identification of vertebral fracture and osteoporosis in lateral spine radiographs and DXA vertebral fracture assessment to predict incident fracture. J Bone Miner Res, 2025. 40(5): p. 628–638.
  - This study demonstrates that automated deep learning–based detection of vertebral fractures from routinely acquired spine imaging provides strong long-term prognostic value for incident fractures. It highlights the clinical utility of integrating fracture detection into opportunistic risk stratification frameworks beyond bone mineral density.
- 54. Kim, Y., et al., A CT-based Deep Learning Model for Predicting Subsequent Fracture Risk in Patients with Hip Fracture. Radiology, 2024. 310(1): p. e230614.
  - This study presents a deep learning–based prognostic model that predicts subsequent fracture risk directly from routine CT images in patients with prior hip fracture. By leveraging end-to-end image representations, it demonstrates how imaging can stratify residual fracture risk in high-risk populations beyond conventional clinical and densitometric measures.
- 56. Chen, K.C., et al., Deep learning meets chest X-rays: a promising approach for predicting future compression fracture risk. Ther Adv Musculoskelet Dis, 2025. 17: p. 1,759,720 × 251,357,157.
  - This study demonstrates the feasibility of predicting future vertebral compression fractures directly from routine chest radiographs using deep learning, with robust external validation. It underscores the potential of widely available non-skeletal imaging for scalable opportunistic fracture screening.

## Data Availability

No datasets were generated or analysed during the current study.
